# OntoStudyEdit: a new approach for ontology-based representation and management of metadata in clinical and epidemiological research

**DOI:** 10.1186/s13326-015-0042-0

**Published:** 2015-12-18

**Authors:** Alexandr Uciteli, Heinrich Herre

**Affiliations:** Institute for Medical Informatics, Statistics and Epidemiology (IMISE), University of Leipzig, Leipzig, Germany

## Abstract

**Background:**

The specification of metadata in clinical and epidemiological study projects absorbs significant expense. The validity and quality of the collected data depend heavily on the precise and semantical correct representation of their metadata.

In various research organizations, which are planning and coordinating studies, the required metadata are specified differently, depending on many conditions, e.g., on the used study management software. The latter does not always meet the needs of a particular research organization, e.g., with respect to the relevant metadata attributes and structuring possibilities.

**Methods:**

The objective of the research, set forth in this paper, is the development of a new approach for ontology-based representation and management of metadata. The basic features of this approach are demonstrated by the software tool OntoStudyEdit (OSE). The OSE is designed and developed according to the three ontology method. This method for developing software is based on the interactions of three different kinds of ontologies: a task ontology, a domain ontology and a top-level ontology.

**Results:**

The OSE can be easily adapted to different requirements, and it supports an ontologically founded representation and efficient management of metadata. The metadata specifications can by imported from various sources; they can be edited with the OSE, and they can be exported in/to several formats, which are used, e.g., by different study management software.

**Conclusions:**

Advantages of this approach are the adaptability of the OSE by integrating suitable domain ontologies, the ontological specification of mappings between the import/export formats and the DO, the specification of the study metadata in a uniform manner and its reuse in different research projects, and an intuitive data entry for non-expert users.

## Introduction

There is a large variety of particular clinical and epidemiological research projects, which typically produce a large amount of data. The data stem from questionnaires, interviews but also from specific findings and from laboratory analyses. Before these data can be collected, the needed metadata must be precisely specified. The metadata include, in the context of this paper:

- The description of all data elements/items (e.g., questions of a questionnaire, measurements of an investigation) by particular attributes (e.g., question text, description of a measurement, unit of measure, data type, codelist);
- The description of a study structure, i.e., the grouping of the items and the description of the corresponding groups by suitable attributes (e.g., title, commentary). These groups may be modules within an assessment, complete assessments (e.g., questionnaires, interviews, physical examinations, laboratory analyses of taken specimen), or assessment groups (e.g., according to study cohorts or to particular data acquisition time points), as well as all items of the study.

The specification of the metadata in particular research organizations must consider certain requirements, e.g., which item attributes are relevant (e.g., name, label, range, data type, format, unit of measure), how the items should be grouped (e.g., module, item group), or which study management software or data entry tools (hereinafter referred to as study software) are used (e.g., OpenClinica [1], ERT [2]).

In this paper we present and discuss a new approach for the ontology-based representation and management of metadata in clinical and epidemiological research, which is demonstrated by the software tool OntoStudyEdit (OSE). The OSE can easily be adapted to the needs of a particular research organization by the use of a suitable domain ontology. Furthermore, it supports and provides an ontology-based configuration of the import/export functions in the desired formats without the necessity to change the source code. The import/export functions need only to be implemented once for a format type (e.g., xml, excel, sql, pdf), and can be configured by an ontology-based definition of mappings between a format type and the domain ontology. This approach has the advantage that the domain experts (e.g., biometrician, data manager, study assistant) can specify the study metadata according to the common usage in a particular research organization by using the respective familiar terminology and without dealing with technical issues. By the provision of import from various sources and export to several formats the differently specified metadata can be represented on the same semantic basis; hence, the once specified metadata can be reused in various research projects and utilized by different study software (or other tools).

## Methods

### Ontology-based representation of metadata

Metadata are used to describe data, hence, they add more precise meaning to data, the semantics of which remains often underspecified. Since the metadata itself must be specified by some formal representation, the meaning of which should be explained, we arrive at an infinite regress, which must be brought to an end by some basic principle. In our approach this infinite regress is blocked by using the top-level ontology General Formal Ontology (GFO) [3]. GFO provides the most basic layer for ontological foundation and represent a well-established semantic basis for modelling metadata. The generic method of reconstruction, or of modelling the domain entities within the framework of a top-level ontology is called the method of ontological reduction. This method as well as the suitability of GFO for modelling metadata were presented in [4].

The OSE is a plug-in for Protégé-Frames [5], which is conceptually based on the notion of a frame. We decided to use Protégé-Frames for our implementation because it supports the generation of data acquisition forms. The forms can easily be adapted, i.e., the slot widgets (input fields like text field, text area, checkbox, combobox) can be selected and arranged in any given layout. Furthermore, the specification of the slot facets (e.g., cardinality, minimum, maximum, default values) allows to elegantly control the quality of the user input (e.g., alert on missing data, prevent false data entries). In summary, the data acquisition forms of Protégé-Frames permit an intuitive data entry for non-expert users, in comparison, e.g., to the OWL version of Protégé.

Frames are formal representational structures, which are exhaustively classified into the following types: classes, slots, facets and individuals [6]. Together with axioms they form the building blocks for Protégé-Frames ontologies. Classes represent concepts related to a domain. Slots represent properties or attributes of classes, whereas facets describe properties of slots. Slots may be attached to frames, and then they describe properties of that frame. A slot, attached to a frame, can have values, which might be constraint by facets. A slot can be attached to a frame as a template slot or as an own slot [6].

The concepts, represented by Protégé classes, are associated in GFO to categories, and the slots attached to a class frame describe properties of that class. A category is defined in GFO as an entity being independent of time and space, that can be instantiated. A category is represented by some symbolic structure, which denotes a meaning, also called intension. The notion of a class - as used, for example, in UML [7], OWL [8], or Frames - captures relevant aspects of categories. Subsequently, we use the term “class” in the sense of a symbolic representation of a category and the term “slot” - as a symbolic representation of a property or a relation. A meta-class in Protégé-Frames corresponds in GFO to a category the instances of which are themselves categories. In Protégé-Frames each class is an instance of a Standard-Meta-Class. In GFO there exists a meta-category, denoted by Category(2), the instances of which are all categories of first order. A category is of first order if all of its instances are individuals. The meta-classes in Protégé correspond to the second-order categories in GFO, which are extensional subcategories of the category Category(2).

### The three ontology method

The OSE is designed and developed according to the three ontology method [9]. This method for developing software is based on the interactions of three different kinds of ontologies: a task ontology (TO), a domain ontology (DO) and a top-level ontology (TLO). The TO is an ontology for the general problem that the software is intended to solve. The DO provides the domain-specific knowledge, whereas the TLO integrates the TO and the DO and is used as the foundation of them. The TLO also provides means for integrating data from different domains. For integrating the TO and the DO we use the TLO GFO because it is sufficiently expressive, in particular, it contains an ontology of categories (that allows categories of higher order), as well as an ontology of properties and attributives [4].

## Results

### The ontological architecture of the OSE

**Fig. 1:**
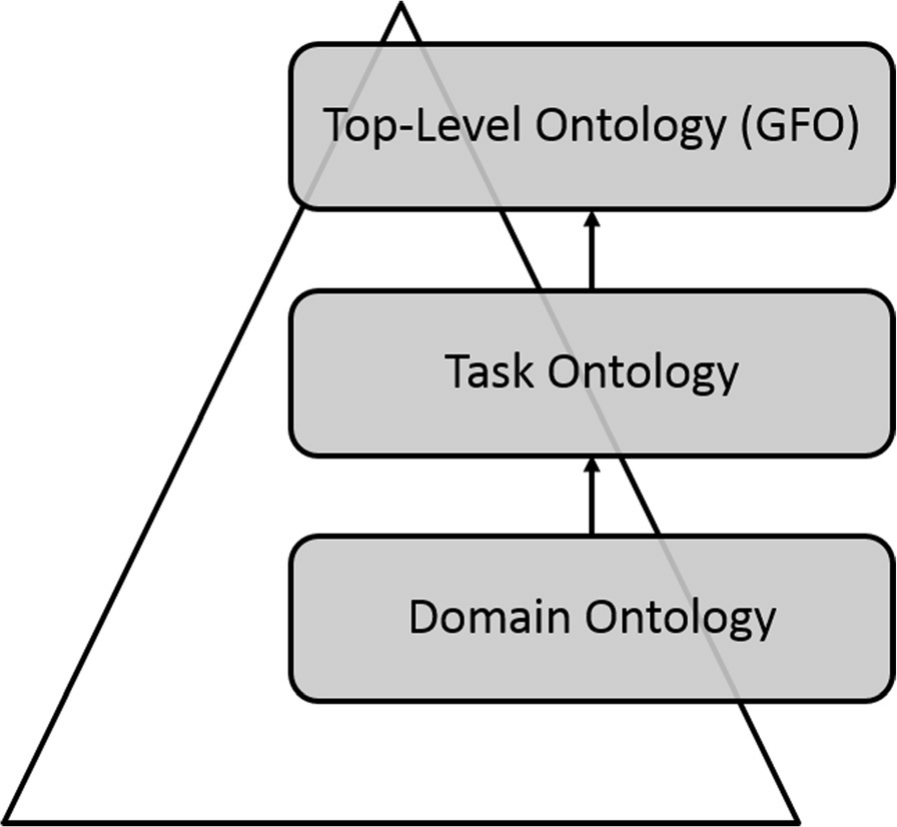
Ontological architecture

The ontological architecture of the OSE is represented by systems of categories of several levels of abstraction and relations between them (Fig. 1). The TO is an upper ontology with respect to the considered DOs, hence, the DO categories are extensional subcategories of the TO categories. The GFO is used as semantic foundation for both TO and DO by classifying categories of TO and DO under particular GFO categories (e.g., category, process, individual).

We use classes for the representation of categories and slots for the representation of properties and relations in Protégé-Frames ontologies.

#### Task and domain ontology of the OSE

**Fig. 2:**
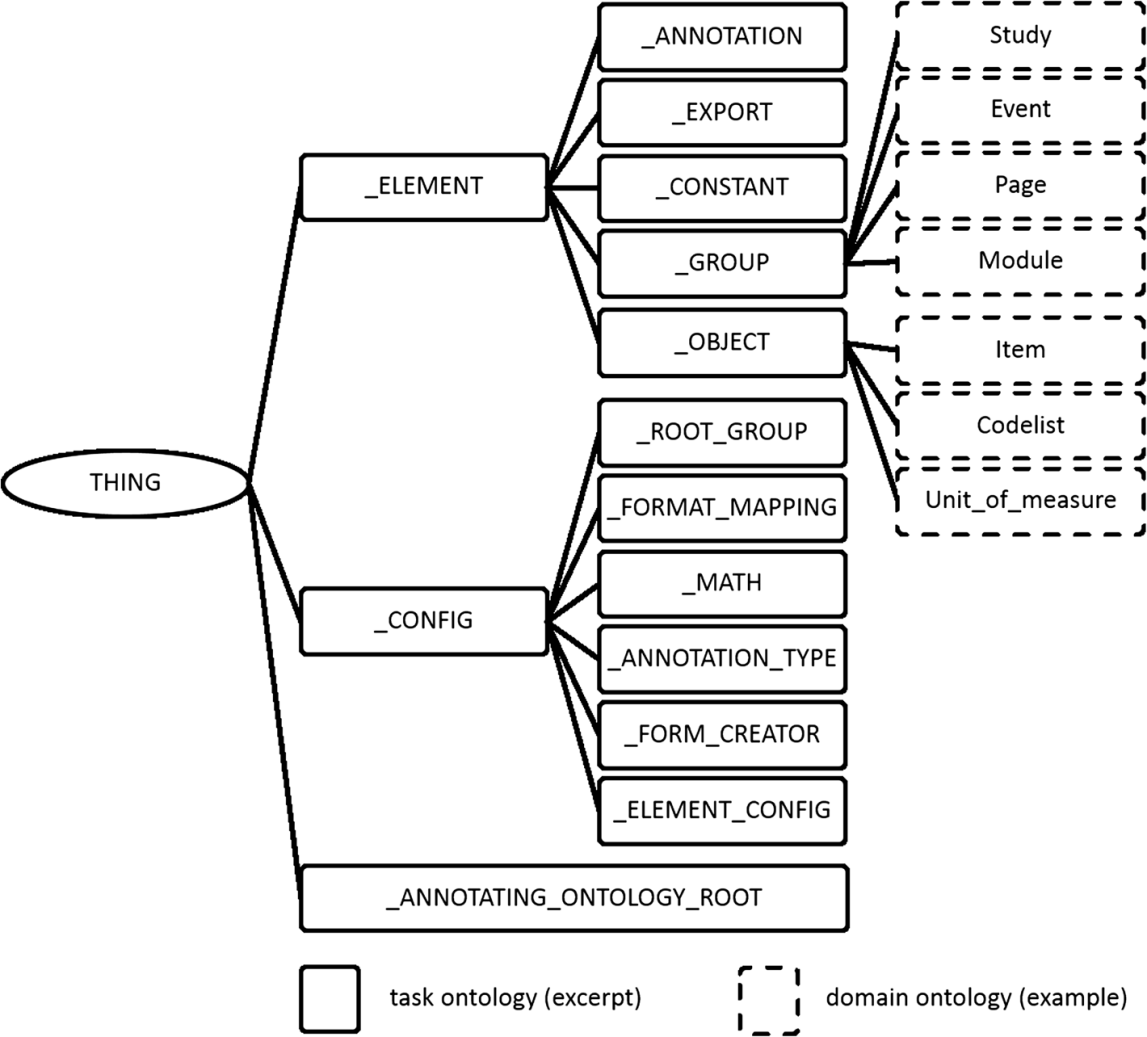
Task and domain ontology

In this section we consider the classes which represent categories of the TO and DO (Fig. 2).

The names of TO classes starts with the underline-sign. The TO includes at the upmost level following classes: _CONFIG, _ELEMENT, and _ANNOTATING_ONTOLOGY_ROOT. The annotating ontologies are inserted below the node _ANNOTATING_ONTOLOGY_ROOT (see Annotating ontologies and annotation).

The class _ELEMENT, its subclasses and instances are visible for the user. The instances can be edited by the user by means of a graphical user interface (GUI). On the other hand, the class _CONFIG, and its subclasses and instances are hidden from the user; these classes and its instances are used by the OSE in the background.

The subclasses of _ELEMENT are: _CONSTANT, _OBJECT, _GROUP, _ANNOTATION, and _EXPORT. The instances of _CONSTANT are used in expressions, the class _OBJECT represents individual entities (e.g., items or measurement units), whereas the class _GROUP stands for lists, which might contain further elements (instances of _ELEMENT). The instances of _ANNOTATION are annotations of elements by concepts of the annotating ontology (see Annotating ontologies and annotation). The subclasses of _EXPORT represent export formats provided for the export function.

The following subclasses of _CONFIG are introduced: _FORMAT_MAPPING, _ANNOTATION_TYPE, the former of which is described in more detail in section Ontological specification of mappings between import/export formats and domain ontology and the latter – in section Annotating ontologies and annotation. In particular, as instances of _ANNOTATION_TYPE various annotation types can be defined. The class _FORMAT_MAPPING and its subclasses are used to specify ontologically various import and export formats.

The class _MATH and its subclasses are used by the expression editor, displayed in the working area E (see Usage of the OSE). The class _MATH_EXPRESSION_RELATOR and its subclasses describe various mathematical operations and functions (for example: AND, >, +). These relators possess arguments, being numbers (_NUMBER), constants (_CONSTANT), particular study elements (_STUDY_ELEMENT, e.g., Item), or further relators. With these means the editor is able to build an expression in form of a tree. The TO includes a number of slots, the most important of which are the following: _contains and _HIERARCHY_SUBCLASS. The _contains slot represents the relation between instances (e.g., Page: B1 _contains Module: Socio-demographic data), whereas the slot _HIERARCHY_SUBCLASS describes the corresponding basic relation between classes (example: Page has _HIERARCHY_SUBCLASS Module). The relation _HIERARCHY_SUBCLASS is formally defined as follows:

Cat_1 _HIERARCHY_SUBCLASS Cat_2 :=

(∀ x y) (x :: Cat_1 ⋀ _contains(x,y) → y :: Cat_2).

A further constituent of the ontological architecture is the DO. This ontology is embedded into the TO, hence, these classes are subclasses of TO classes. We developed an example DO, based on the structure of clinical trials which are carried out at the Clinical Trial Centre Leipzig [10]. Classes like Study or Module are placed below the class _GROUP, whereas Item or Unit_of_Measure are subclasses of _OBJECT. The slots of these classes can freely be defined. The class Item, for example, can possess following slots: name, description, unit_of_measure, range, codelist or rules.

#### Ontological specification of mappings between import/export formats and domain ontology

In this section we outline the specification of mappings between import/export formats and DO for the example of an xml-based format, CDISC ODM [11]. For the mapping specification the subclasses of _FORMAT_MAPPING are used.

**Fig. 3:**
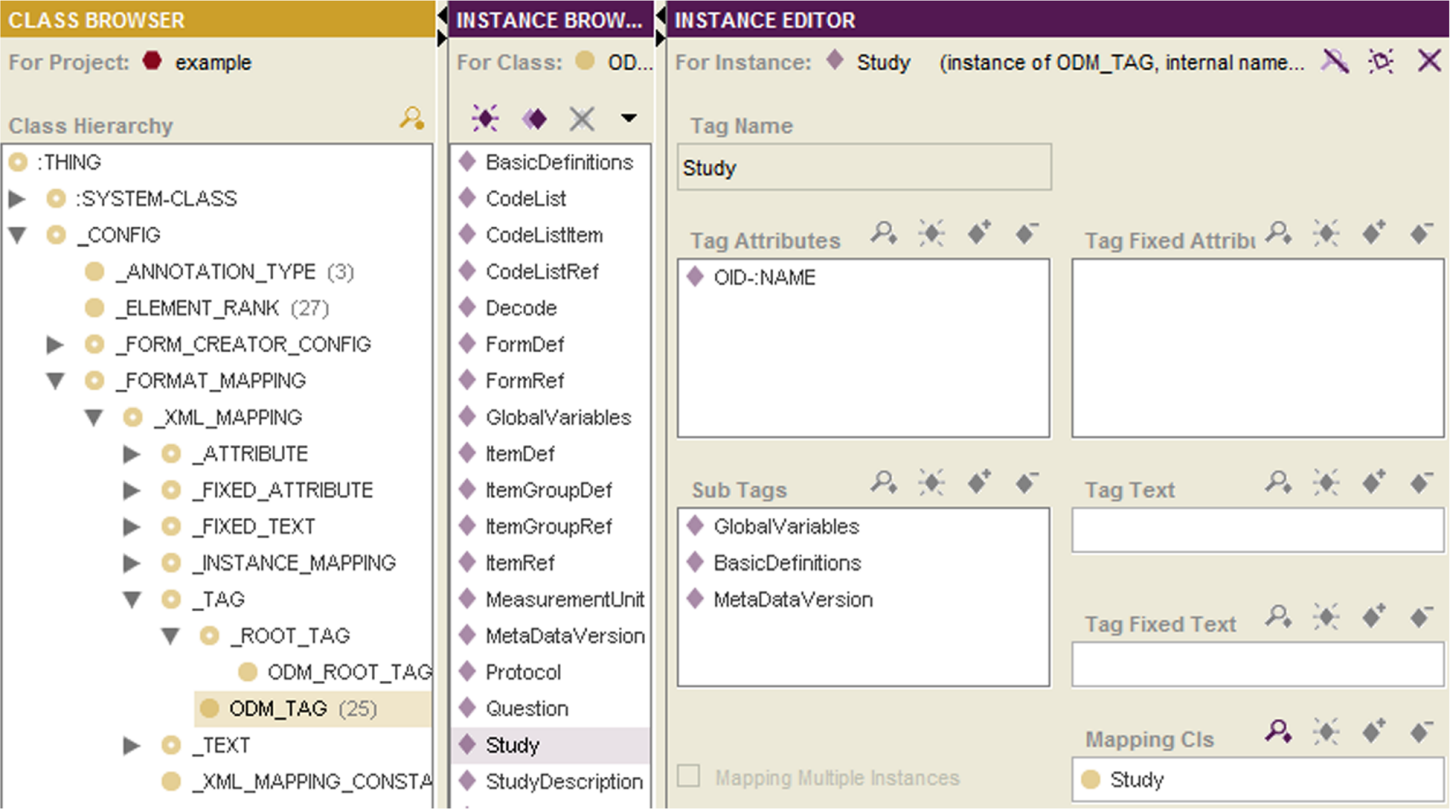
ODM format mapping

The tag structure of an xml-based format can be considered as a tree. As a first step the root tag (in our example, <ODM>) must be specified as instance of _ROOT_TAG. The other tags are defined as instances of _TAG. For each tag its sub-tags and attributes must be specified by the instance editor (Fig. 3). The tag <Study> contains, e.g., the sub-tags <GlobalVariables>, <BasicDefinitions>, and <MetaDataVersion> as well as the attribute “OID”. The tags are mapped to the DO classes, whereas their texts and attributes - to the DO slots. In our example, the tag <Study> is mapped to the class Study and the attribute “OID” - to the slot :NAME of the class Study.

The whole tag tree must be specified only once; this is done by declaration of the corresponding sub-tags and attributes and by the specification of its mapping to the classes resp. slots of the DO. Then, this mapping can be used for the import/export of various metadata specifications.

#### Annotating ontologies and annotation

By using Protégé it is possible to import an ontology into another one. We could, e.g., import the ACGT Master Ontology [12], phenotype or property ontologies, or LOINC [13] and may use their categories for the annotation of the instances of the DO (e.g., concrete item instances like DYSPNEA_AT_REST). Ontologies, intended to be used to annotate instances of a DO within the OSE, are called in this paper annotating ontologies.

For this purpose, we introduced the following classes into the TO:

- _ANNOTATION_TYPE (subclass of _CONFIG). Within DO we may define various annotation types, being instances of _ANNOTATION_TYPE (e.g., annotated_with, risk_factor_of, symptom_of).
- _ANNOTATION (subclass of _OBJECT). This class has three slots: _annotated_elements, _annotation_type und _annotating_concepts. Using OSE we may create concrete annotations as instances of _ANNOTATION. This is realized by selecting the elements to be annotated, taken from subclasses of _ELEMENT, by choosing an annotation type from the instances of _ANNOTATION_TYPE, and by selecting suitable classes taken from the annotating ontology.
- _ANNOTATING_ONTOLOGY_ROOT. Below this class annotating ontologies can be inserted.
- _ANNOTATING_ONTOLOGY_METACLASS (subclass of :STANDARD-CLASS). This is a meta-class, containing all classes of the annotating ontology as instances. This class has an additional slot, denoted by _annotations, which is defined as the inverse slot of _annotating_concepts.

**Fig. 4:**
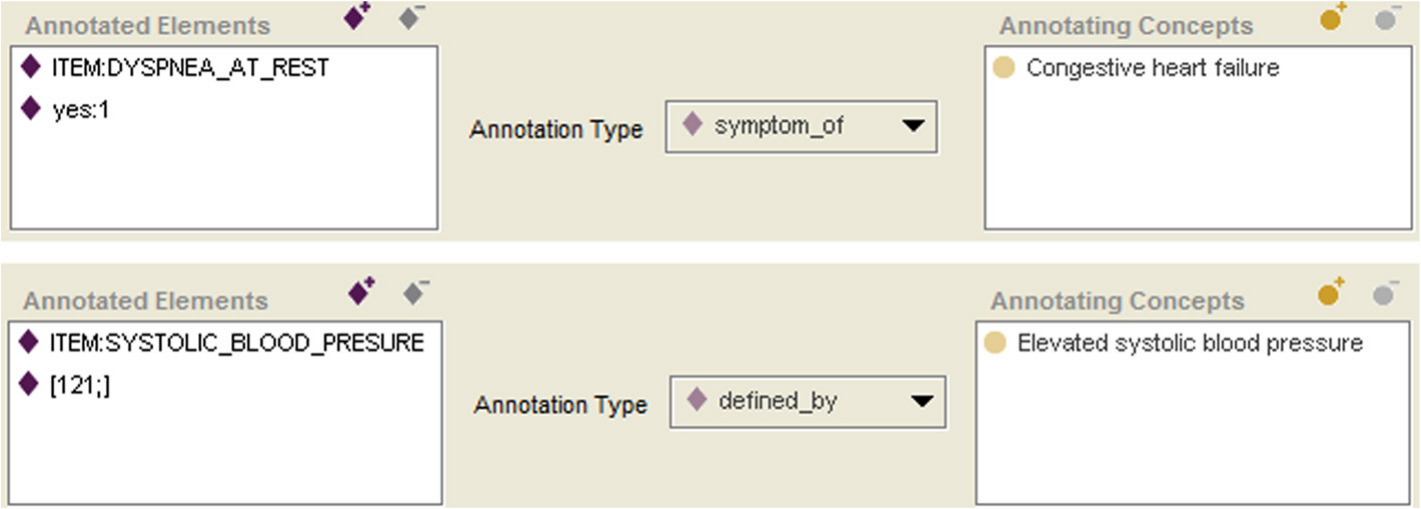
Annotation instance (examples)

We may not only annotate single instances, but also sets of instances. It is, e.g., not sufficient to annotate the item ITEM:DYSPNEA_AT_REST as a symptom of „Congestive heart failure“, taken from the Human Phenotype Ontology (HPO) [14]. This item is associated with a codelist that includes two possible values “YES” or “NO” (depending on whether a patient has dyspnea or not). Only if “YES” is selected as answer to the question whether “dyspnea at rest” holds, this symptom is true. I.e., only the combination of the item ITEM:DYSPNEA_AT_REST and the answer option “YES” can be annotated (Fig. 4). In this way both conditions are AND-connected, expressing that the question was answered and that the answer is “yes”. If an item does not possess a codelist, then also ranges can be annotated. An example is the annotation of the item ITEM:SYSTOLIC_BLOOD_PRESURE together with Range [121;] (i.e., >= 121) with the concept „Elevated systolic blood pressure“ from HPO (Fig. 4).

**Fig. 5:**
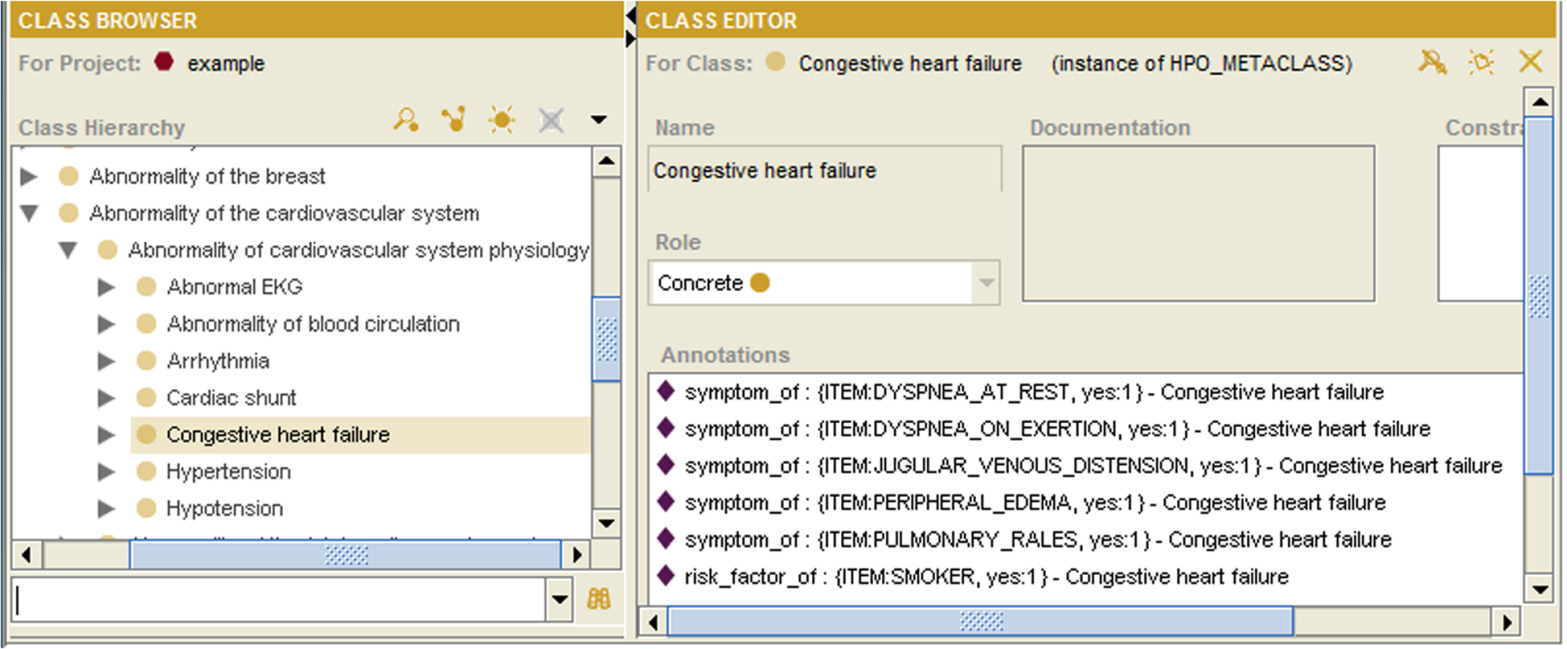
Concept annotations (example)

By use of the inverse slot _annotations for classes of the annotating ontology we may realize the following valuable feature: for a given concept all of its annotations can be displayed. This functionality can be very important in searching for items in certain domains during the planning phase of a study. If we are planning, e.g., a study for heart failure we may ask for all annotations of the class „Congestive heart failure“ (and possibly of its subclasses). These annotations will then be displayed. In this way one has a quick access to items which can be used to query the symptoms or risk factors of the heart failure (Fig. 5).

The annotation of different instances of the domain ontology (even of different domain ontologies) by the same class of the same annotating ontology establishes a semantic connection between these instances. Such annotations allow the semantic search for items over the categories, which are used in these annotations, but they also allow the comparability of data which are acquired for various distinct studies.

### Usage of the OSE

Subsequently, we sketch the graphical user interface (GUI) and the main functions of the OSE: specification, management, import and export of metadata, searching and navigation.

**Fig. 6:**
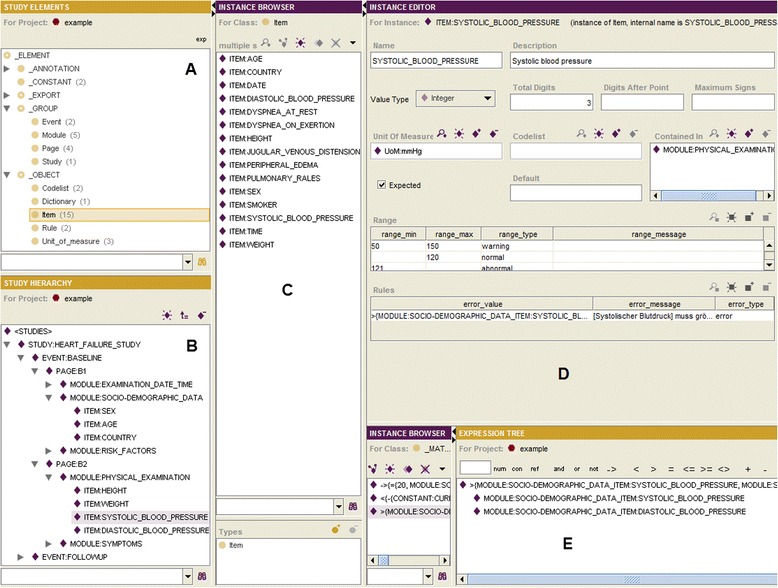
GUI of the OSE. **a** Study Elements; **b** Study Hierarchy; **c** Instance Browser; **d** Instance Editor; **e** Expression Editor

**Fig. 7:**
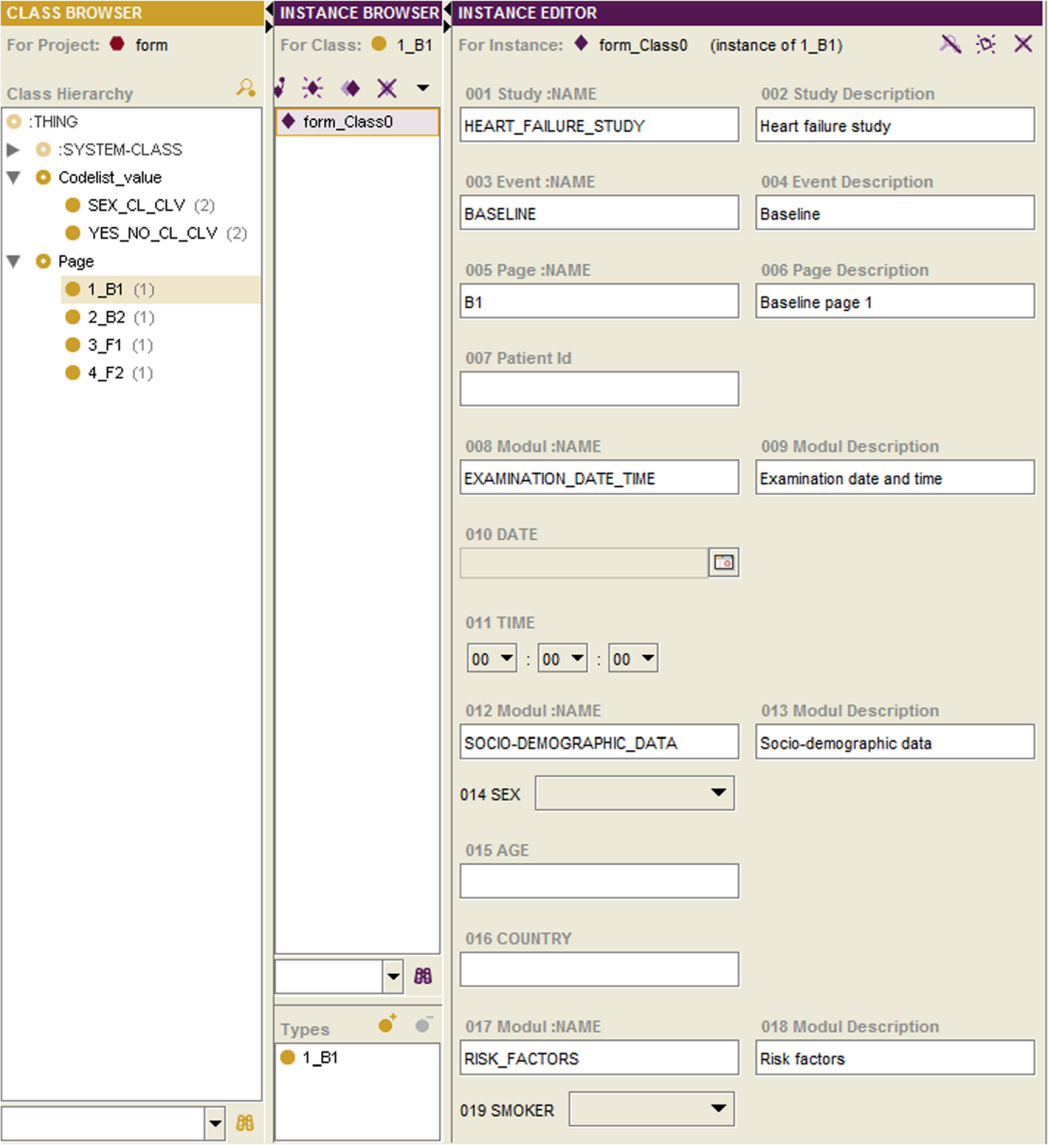
Case report form preview (example)
- B: Study Hierarchy. This hierarchy shows the structure of a study. This hierarchy is formed by instances which are connected by the contains-relation. The user may create new elements in a group, may change the elements’ order, and may remove elements from a group. By choosing an element, a form for the acquisition of its slot values is displayed in the working area D (Instance Editor). Furthermore, a search field is available.
- C: Instance Browser. The instance browser shows instances of the class which is selected in the working area A. By choosing an instance, a form for capturing its slot values will be shown in the working area D (Instance Editor). Instances may be deleted. Furthermore, it is possible to associate instances from this working area to groups from the working area B by drag-and-drop. A search field is available.
- D: Instance Editor. The instance editor provides forms for capturing the slot values of instances.
- E: Expression Editor. This editor supports the editing of formulas, being represented in form of trees. Various operators and numbers can be used, for example arithmetical, logical operators, and other relations; furthermore, study elements (for example items) and constants can be referenced.

The GUI is partitioned into five working regions A, B, C, D and E (Fig. 6):

- A: Study Elements. Within this region all study elements (being subclasses of _ELEMENT) are represented and displayed. Besides a class, the number of its instances is shown (put in brackets). For a selected class its instances are displayed in the working field C (Instance Browser). Furthermore, a searching field is available. For the export of the specified metadata an export format must be selected (a subclass of _EXPORT) and the button “exp” pressed. It is also possible to export a metadata specification as an ontology that can be used as a Case Report Form (CRF) preview (Fig. 7).

The functionalities of the working areas A: Study Elements, B: Study Hierarchy and E: Expression Editor were implemented in the OSE plug-in, whereas the functionalities of the working areas C: Instance Browser and D: Instance Editor are already provided by Protégé as standard features. The working area A was implemented in such a way that only the subtree of _ELEMENT is displayed for the user, whereas the subtree of _CONFIG remains hidden. In this way we may assure that the user of OSE (e.g., data manager, researcher, study assistant) cannot change the configuration that was specified, e.g., by ontologists or IT-specialists. Furthermore, we implemented for this working area the import/export of the metadata specification. The working area B displays the study hierarchy as a tree which connects the instances (i.e., the study elements, as for example, module or item) by the contains-relation, whereas the standard trees of Protégé represent class hierarchies, based on the is-a relation. Moreover, we implemented all functions, needed for manipulating the study structure. Additionally, the expression editor (E) was implemented; this editor provides all needed functions for designing and managing of formulas. We increased the usability of the tool by adapting the automatically generated data acquisition forms (i.e., the slot widgets were replaced and the form layout was changed).

## Related work

There are few systems that pursue similar purposes as OSE, notably TIM (Trial Item Manager) [15] and ObTiMA (Ontology-based Managing of Clinical Trials) [16], which are subsequently considered in more detail.

ObTiMa is a system for ontology-based management of clinical trials, which is composed of the two components: “the Trial Builder for designing clinical trials and the Patient Data Management System for handling patient data within a trial.” Trial Builder allows the creation of CRF items, based on the concepts of an ontology (ACGT Master Ontology). The main difference between the ObTiMa’s Trial Builder and the OSE consists in that the Trial Builder is based on ODM and the possible item attributes (e.g., question, data type, measurement unit) are fixed and cannot be changed or extended, whereas in OSE item attributes are defined by domain ontologies and can be flexibly handled. Hence, OSE may take into account the needs of the diverse research organizations, which usually differs with respect to the practiced specification of metadata that typically use different metadata types (e.g., items, codelists, modules), different attributes, groupings, and hierarchical levels (e.g., study-event-module-item). Furthermore, the flexible, ontology-based development of mappings between the ontologies of OSE and diverse import and export formats enables the reuse of specified metadata in various research projects and their utilization by different study software.

The TIM pursues aims, analogous to OSE, namely, to support the specification of items in clinical trials. Similarly as OSE, TIM is based on a semantic model consisting of a fixed component (the meta-model and the core types of the data model), and a flexible module (domain-specific types of the data model). This structure supports the adaption to user-specific needs. Though, there are differences between TIM and OSE. In TIM the fixed component and the flexible part are not clearly separated, whereas in OSE both components (TO and DO) are explicitly divided and endowed with an ontologically-based semantic basis. Consequently, OSE exhibits a higher flexibility with respect to the change and adaption of the domain-specific constituents. Furthermore, OSE provides various additional functionalities, among them, the ontologically-based creation of format mappings, and the use of rule expressions. Finally, the usage of Protégé-Frames supports the adaption of the data acquisition forms, and allows for an extension of the software by additional plug-ins.

## Conclusions and future work

In this paper we presented and discussed a new approach for ontology-based representation and management of metadata in clinical and epidemiological research using the software tool OntoStudyEdit (OSE). Advantages of this approach are: 1. the adaptability of the OSE to intended aims and given needs by integrating suitable domain ontologies in a modular way; 2. the ontological specification of mappings between the import/export formats and the DO, such that no changes of the source code are needed by the replacement of the DO; 3. the specification of the study metadata in a uniform manner and reuse of which in different research projects; 4. an intuitive data entry for non-expert users.

The OntoStudyEdit is a tab widget plug-in for Protégé-Frames; this implies that all functionalities of Protégé can be used. Of particular interest is the adaption of the data acquisition forms. At present, we are working on the implementation of further import/export functions, e.g., related to annotated CRF in PDF format and to specifications for the import in different study software.

There is ongoing evaluation of the OSE which started already some time ago. At first, metadata of the LIFE study are entered with the OSE. For LIFE we developed an ontology, called LIFE Investigation Ontology (LIO), and a Protégé-Frames based tool, called query generator [17]. The metadata part of LIO was integrated into OSE as a domain ontology. LIO is a frame ontology and its use as domain ontology in OSE preserves the structure of the LIFE metadata. For this reason it is rather simple to input and to manage the LIFE metadata by using the OSE. We already experienced that the non-expert users (e.g., data manager, researcher, study assistants) are able to cope well with OSE, as well as with the LIFE query generator, that is productively used since two years by the same user group.
